# PIM2 interacts with tristetraprolin and promotes breast cancer tumorigenesis

**DOI:** 10.1002/1878-0261.12192

**Published:** 2018-04-14

**Authors:** Chune Ren, Tingting Yang, Pengyun Qiao, Li Wang, Xue Han, Shijun Lv, Yonghong Sun, Zhijun Liu, Yu Du, Zhenhai Yu

**Affiliations:** ^1^ Department of Reproductive Medicine Affiliated Hospital of Weifang Medical University Shandong China; ^2^ Department of Pathology Affiliated Hospital of Weifang Medical University Shandong China; ^3^ Department of Medical Biology Weifang Medical University Shandong China

**Keywords:** cancer, cell proliferation, degradation, proviral insertion in murine lymphomas 2, tristetraprolin

## Abstract

Tristetraprolin (TTP) is an AU‐rich element‐binding protein that regulates mRNA stability and plays important roles in cancer. The mechanisms by which TTP is regulated in breast cancer are poorly understood. Using multiple biochemical approaches, we found that proviral insertion in murine lymphomas 2 (PIM2) is a novel binding partner of TTP. Interestingly, PIM2 decreased TTP protein levels independent of its kinase activity. PIM2 instead targeted TTP protein for degradation via the ubiquitin‐proteasome pathway. Furthermore, immunohistochemical staining showed that PIM2 and TTP protein levels were negatively correlated in human breast cancer samples. Indeed, PIM2 overexpression de‐repressed TTP‐mediated inhibition of breast cancer cell proliferation and migration *in vitro* and promoted breast tumor xenograft growth *in vivo*. These findings demonstrate an important role for the PIM2‐TTP complex in breast cancer tumorigenesis, suggesting that PIM2 may represent a potential therapeutic target for breast cancer treatment.

**Abbreviations**
AR: androgen receptor (AR)
Co‐IP: co‐immunoprecipitation cycloheximide (CHX)
DMEM: Dulbecco's modified Eagle medium
ER: estrogen receptor
GR: glucocorticoid receptor
GST: glutathione S‐transferase
H&E: hematoxylin & eosin
HER2: human epidermal growth factor receptor 2
IHC: immunohistochemistry
IP: immunoprecipitation
NF‐kB: nuclear factor kappa B
PIM2: Proviral Insertion in Murine Lymphomas 2
PR: progesterone receptor
PVDF: poly(vinyl)idene difluoride
TTP: tristetraprolin

## 1 Introduction

Tristetraprolin (TTP) is an AU‐rich element‐binding protein that initiates mRNA decay and is found in all major eukaryotes (Patial and Blackshear, 2016). Human TTP belongs to the TIS11 gene family, which is composed of three genes: *ZFP36* (TTP), *ZFP36L1* and *ZFP36L2*. TTP suppresses the inflammation response by destabilizing proinflammatory mRNAs including c2, interleukin (IL)‐6, IL‐8, IL‐10, IL‐13, IL‐16, IL‐17, IL‐22, IL‐23 and IL‐33 (Khabar, 2017). In addition to its functions in immune response, TTP plays an important role in tumorigenesis. Compared with normal tissues, TTP expression is low in many types of cancer, including breast, thyroid, lung, ovary and cervical cancers, and low TTP expression in those diseases is associated with poor patient survival (Griseri and Pages, 2014). Moreover, TTP targets various oncogene‐encoding mRNAs to delay tumor progression. For example, TTP regulates the mRNA stability of c‐Myc (Marderosian *et al*., 2006) and HIF‐1α (Chamboredon *et al*., 2011) to inhibit tumor‐cell proliferation. TTP is phosphorylated by multiple kinases including PKA, AKT, CK2, p38 MAPK, MK2, JNK, ERK1 and MEKK1, which are crucial in the regulation of its functions (Brooks and Blackshear, 2013; Clark and Dean, 2016). TTP expression is deficient in breast cancer (Pandiri *et al*., 2016). Therefore, the induction or overexpression of TTP may lead to new strategies for treating breast cancer. But the mechanisms of TTP protein degradation are still unclear.

PIM2 was first identified as a provirus integration site for Moloney murine leukemia virus, which belongs to a serine/threonine kinase family encoded by proto‐oncogenes including PIM1 and PIM3 (Warfel and Kraft, 2015). PIM2 is highly conserved, with >60% sequence identity to PIM1 and PIM3 (Jinesh *et al*., 2016). PIM2 lacks a regulatory domain and thus is constitutively activated, a trait which is used in the design of tumor drugs (Paino *et al*., 2017). PIM2 plays an important role in multiple types of cancer; it is frequently overexpressed in human cancers and promotes cancer‐cell proliferation and survival (An *et al*., 2015). PIM2 as a kinase could phosphorylate a series of proteins that are essential for tumor progression (Le *et al*., 2015). For example, PIM2 targets the TSC2 protein and enhances the mTOR‐C1 pathway to maintain cell growth in multiple myeloma, representing an important target in the treatment of tumor progression and bone loss in myeloma (Hiasa *et al*., 2015; Lu *et al*., 2013). Moreover, FOXP3 is phosphorylated by PIM2, which influences the stability and function of regulatory T cells (Deng *et al*., 2015). PIM2 also functions independently of its kinase activity as a co‐factor that augments the transcriptional activity of HIF‐1α (Yu *et al*., 2014b). Breast cancer is the most common cancer in women and the second leading cause of cancer death, and it is reported that PIM2‐expressing transgenic mice induce breast hyperplasia and tumors (Jimenez‐Garcia *et al*., 2017). However, the mechanisms by which PIM2 regulates breast cancer remain uncharacterized.

Here, we show that PIM2 is a novel binding partner for TTP. PIM2 interacts with TTP *in vivo* and *in vitro*. PIM2 negatively regulates TTP protein levels independently of its kinase activity. Moreover, PIM2 promotes TTP protein degradation through the ubiquitin proteasome pathway. As a result, PIM2 stimulates TTP‐mediated proliferation and migration in breast cancer cells. Interestingly, PIM2 expression is positively correlated with tumor stage and size in breast cancer, and knockdown of PIM2 increases TTP protein levels *in vivo*, which promotes breast tumor growth. Our findings provide new insights into the mechanisms underlying oncogenetic functions of PIM2 in breast cancer and may present new therapeutic strategies for breast cancer treatment.

## 2 Materials and methods

### 2.1 Cell culture

HEK293T, MCF‐7 and MDA‐MB231 cell lines were cultured in Dulbecco's modified Eagle medium (DMEM) supplemented with 10% FBS (Gibco, Carlsbad, CA, USA) as well as 100 μg·mL^−1^ penicillin and 100 μg·mL^−1^ streptomycin at 37 °C and 5% CO_2_.

### 2.2 SiRNA and transfection

Small interfering (si) RNAs against PIM2 and TTP were purchased from Shanghai GenePharma (Shanghai, China). All transient transfections were performed in six‐well plates using Lipofectamine 3000 (Invitrogen, Shanghai, China) according to the manufacturer's instructions. After 24 h, cells were re‐plated in the indicated size plates to perform other experiments. The total amounts of plasmids were 2 μg per well, and siRNA were 100 pmol per well. The siRNA sequences are listed in Table S2.

### 2.3 DNA constructs and mutagenesis

PCR‐amplified human genes used in this study were cloned into pcDNA3.0/HA (N‐), pFlag‐CMV4, pEGFP‐C1, pet28a‐His or pGEX‐4T‐1. The mutants were generated using QuickChange site‐directed mutagenesis kit (Stratagene, Beijing, China). The PIM2 shRNA was generated with oligonucleotide 5‐CTCGAAGTCGCACTGCTAT‐3 and the lentiviruses were produced by GenePharma.

### 2.4 Quantitative real‐time PCR

Total RNA was isolated by TRIzol kit (Omega), and cDNA was synthesized by the cDNA synthesis kit (Takara, Dalian, China). Quantitative real‐time PCR was performed using the SYBR Green PCR Master Mix (Takara) with the CFX96 Real‐Time PCR detection system (Bio‐Rad, Shanghai, China). Primer sequences are listed in Table S2.

### 2.5 Western blot

Cells were harvested and lysed with ice‐cold lysis buffer (Beyotime, Shanghai, China, P0013). After centrifugation at 12 000 ***g*** for 10 min at 4 °C, proteins in the supernatants were quantified and boiled 10 min at 100 °C with the 5× SDS sample buffer, separated by SDS‐PAGE and transferred to poly(vinyl)idene difluoride (PVDF) membranes. After blocking, membranes were immunoblotted with the indicated antibodies. Immunoreactivity was detected with enhanced chemoluminescent autoradiography according to the manufacturer's instructions. The antibodies used are listed in Table S2. The intensities of immunoblotting analyses were measured using imagej software (National Institutes of Health, Bethesda, MD, USA).

### 2.6 Co‐immunoprecipitation (Co‐IP) and glutathione S‐transferase (GST) pull‐down assays

GST or His fusion proteins were expressed in *Escherichia coli* BL21 (DE3) and purified. Co‐IP and GST pull‐down assays were performed as described previously (Yu *et al*., 2013, 2014a).

### 2.7 Confocal immunofluorescence microscopy

Cells were re‐plated into six‐well plates with a density of 1 × 10^5^ cells per well. Confocal immunofluorescence microscopy was performed as described previously (Yu *et al*., 2013, 2016).

### 2.8 *In vitro* kinase assay

GST‐TTP or GST‐PKM2 1 μg recombinant proteins were incubated with 0.2 μg His‐PIM2 in 50 μL kinase buffer. The reaction mixtures were incubated at 37 °C for 30 min. Aliquots of reaction mixtures were analyzed by western blotting using indicated antibodies (Yu *et al*., 2013).

### 2.9 Cell proliferation analysis

Cells were seeded onto six‐well plates, transfected with indicated plasmids. After 24 h, 2 × 10^4^ cells were harvested and seeded in triplicates onto new 24‐well plates, and cell numbers were counted every 24 h over a 4‐day period.

### 2.10 Clone formation experiment

Cells were seeded onto six‐well plates at a concentration of 500 cells in 2 mL culture medium per well and cultured at 37 °C in 5% CO_2_ for 12–14 days. The culture medium was removed and 1 mL 4% paraformaldehyde was used to fix the cells at room temperature for 15 min. After removing the fixative solution, the cells were treated with crystal violet staining solution for 20 min, washed with ddH_2_O, and cells photographed.

### 2.11 Wound healing assay and cell invasion assay

Cells were seeded in six‐well plates that were incubated in culture medium until a monolayer was formed. The monolayer was then wounded by scratching with pipette tips and washed with PBS. After 24 h, cells were photographed at 100× magnification under a light microscope. Cell invasion assay were performed as described previously (Yu *et al*., 2014a).

### 2.12 Tissue collection and immunohistochemistry

This study was approved by the Ethics Committee of the Weifang Medical University. We studied 10 cases of normal breast tissues and 84 breast cancer tissues. All patients provided their full consent to participate in the study at the Affiliated Hospital of Weifang Medical University (Weifang, China). The histopathological parameters including estrogen receptor (ER), progesterone receptor (PR) and human epidermal growth factor receptor 2 (HER2) were retrieved from the patients’ pathology reports. PIM2 expression was detected in all specimens. The sections were dewaxed with xylene followed by rehydration in graded alcohol. Antigen retrieval was carried out for 30 min by heating in a microwave oven. Endogenous peroxidase activity was then blocked by incubating the sections with 3% H_2_O_2_ for 20 min; nonspecific staining was blocked by incubating with 10% normal bovine serum for 1 h. Sections were incubated with antibody against PIM2 or TTP at 4 °C overnight and then incubated with the HRP‐conjugated secondary antibody. The signals were visualized with diaminobenzidine as the chromogen and counterstained by hematoxylin. Sections were examined separately by two pathologists under double‐blinded conditions without prior knowledge of the clinical status of the sections. The percentage of positive staining was graded as 0 = 0–5%, 1 = 6–25%, 2 = 26–50%, 3 = 51–75% and 4 = 76–100%, with a staining intensity score of 0 = none, 1 = weak, 2 = moderate and 3 = strong. The staining grade was stratified as absent (score 0), weak (score 1–4), moderate (score 5–8) or strong (score 9–12). Tumors with a score of > 4 were classified as having high expression and tumors with a score of ≤ 4 as having low expression. The antibodies and kit used are listed in Table S2.

### 2.13 Xenograft studies in nude mice

All experimental protocols were approved by the Ethic Committee for Animal Experimentation of Weifang Medical University. Sixty‐day release pellets containing 17β‐estradiol (0.18 mg) (Innovative Research of America, Sarasota, FL, USA) were implanted subcutaneously 2 days before injecting the cells. The viruses were infected into MCF‐7 cells to form stable knockdown of PIM2. MCF‐7 cells 5 × 10^6^ were injected subcutaneously into either the right or left shoulder sides of BALB/c nude mice (4‐week‐old females). Tumor volumes were measured during the tumor growth for 3 weeks. Tumor volumes were calculated according to the following formula: Tumor volume = (length × width^2^)/2. After 3 weeks, the mice were killed and tumors were weighed prior to further histological evaluation.

### 2.14 Statistical analysis

All data represented the mean ± SD of at least three independent experiments. Sample number (*n*) indicated the number of independent biological samples in each experiment. Sample numbers and experimental repeats are indicated in figure legends. All statistical analyses were done using spss software version 17.0 (Chicago, IL, USA). Two‐tailed *t*‐tests were used to analyze the difference between the two groups. *P* < 0.05 was considered to be significant.

## 3 **Results**

### 3.1 PIM2 interacts with TTP

**Figure 1:**
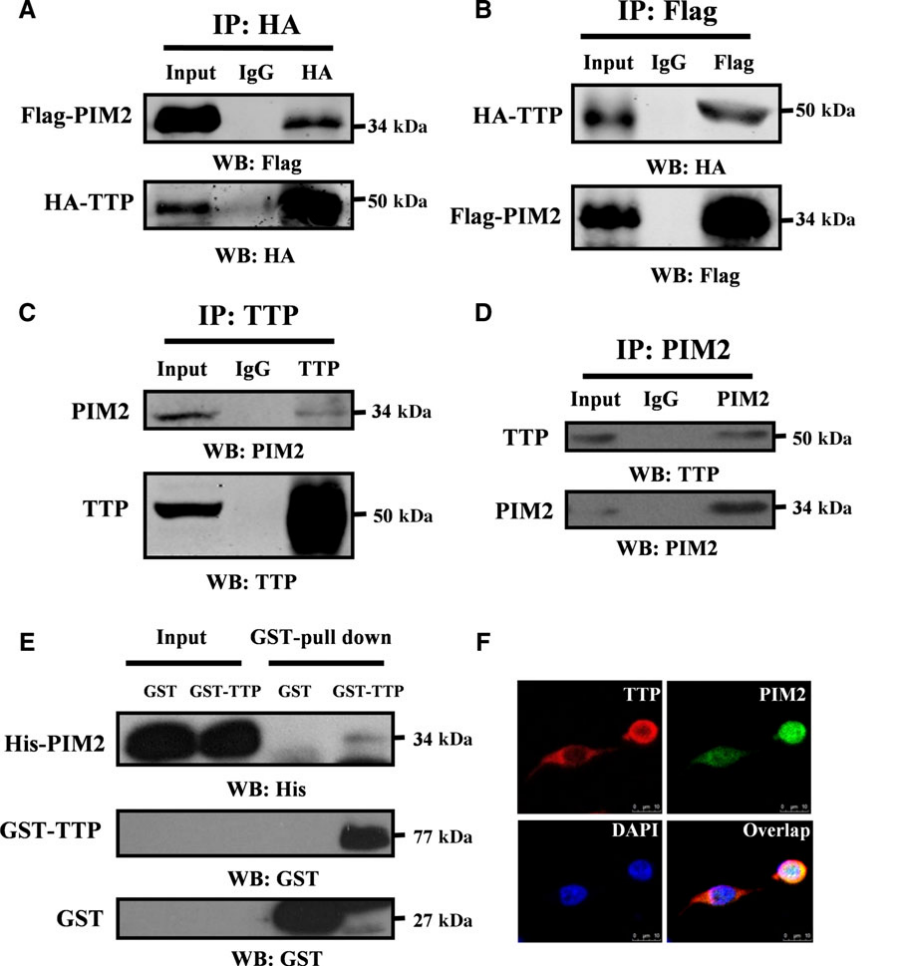
PIM2 interacts with TTP. (A, B) HEK293T cells were co‐transfected with HA‐tagged TTP and Flag‐tagged PIM2. After 24‐h culture, cell lysates were prepared in IP buffer and HA‐tagged proteins were immunoprecipitated with anti‐HA antibody (A) or anti‐Flag antibody (B) (IgG as control). The presence of Flag‐ or HA‐tagged proteins was examined by western blotting using anti‐HA or anti‐Flag antibody. (C, D) Co‐IP of endogenous PIM2 and TTP from MCF‐7 cells using anti‐TTP (C) or PIM2 (D) as the antibodies (IgG as a negative control). The presence of PIM2 or TTP proteins in IP elutes was examined by western blotting using anti‐PIM2 or anti‐TTP antibody. (E) Recombinant GST‐tagged TTP or GST alone proteins were incubated with recombinant His‐tagged PIM2 overnight. Anti‐His antibody was used to detect His‐tagged PIM2 proteins in elutes. (F) Confocal immunofluorescence microscopy was performed to analyze the location of TTP and PIM2 in MCF‐7 cells.

Most of the previous studies of TTP have focused mainly on the functions of TTP in breast tumor progression (Barrios‐Garcia *et al*., 2014; Milke *et al*., 2013; Pandiri *et al*., 2016). The regulatory signaling factors that directly regulate TTP are not fully defined. To search for new interacting partners of TTP, we performed immunoprecipitation (IP) assays in MCF‐7 cells using HA‐tagged TTP as a bait. The identities of binding proteins were determined by mass spectrometry. Unexpectedly, we found that PIM2 was a novel binding partner of TTP (Table S1). To further analyze their interaction, we transiently overexpressed HA‐tagged TTP and Flag‐tagged PIM2 in HEK293T cells. By Co‐IP analysis, we found that PIM2 could bind to TTP (Fig. 1A,B). To determine whether endogenous TTP interacts with endogenous PIM2, we performed Co‐IP experiments with cell lysates from MCF‐7 cells. Using an anti‐TTP antibody, we showed that endogenous TTP could co‐immunoprecipitate endogenous PIM2 (Fig. 1C). Likewise, endogenous PIM2 could co‐immunoprecipitate endogenous TTP (Fig. 1D). Furthermore, to determine whether PIM2 can interact directly with TTP, we performed a GST) pull‐down assay. We purified recombinant GST‐tagged TTP proteins from bacteria and incubated them in the presence of glutathione‐sepharose beads with His‐tagged PIM2. As shown in Fig. 1E, the GST‐tagged TTP strongly bound to the His‐tagged PIM2. Furthermore, immunofluorescence confocal microscopy showed that the endogenous TTP and PIM2 mostly overlapped in the cytoplasm of the MCF‐7 cells but only partially overlapped in the nucleus of those cells (Fig. 1F). Taken together, our results indicate that TTP interacts physically with PIM2.

### 3.2 PIM2 binds to the tandem zinc finger domain of TTP

**Figure 2:**
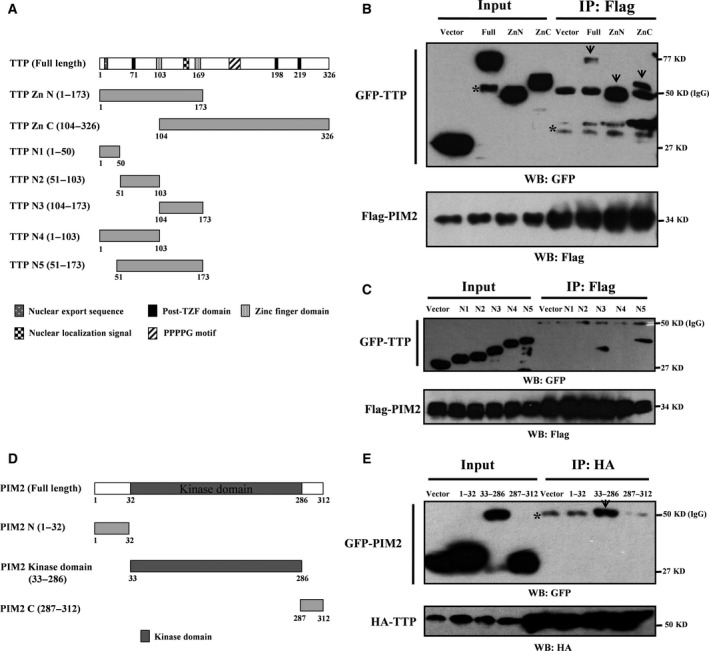
PIM2 binds to tandem zinc finger domain of TTP. (A) TTP truncation mutants used in this study. (B, C) MCF‐7 cells co‐transfected with Flag‐tagged PIM2 and GFP‐tagged TTP fragments as indicated. Co‐IP assay followed by western blotting was performed to determine their interaction (* unspecific band). (D) PIM2 truncation mutants used in this study. (E) MCF‐7 cells co‐transfected with HA‐tagged TTP and GFP‐tagged PIM2 fragments as indicated. Co‐IP assay followed by western blotting was performed to determine their interaction (*unspecific band).

To identify the domain(s) of TTP required for interaction with PIM2, we generated two TTP fragments: GFP‐tagged N‐terminal truncation ZnN (1–173aa) and GFP‐tagged C‐terminal truncation ZnC (104–326aa), both of which had a tandem zinc finger domain (Fig. 2A). We co‐expressed the GFP‐tagged TTP and Flag‐tagged PIM2 in MCF‐7 cells and performed Co‐IP assays. As shown in Fig. 2B, PIM2 strongly bound to the N‐terminal domain of TTP but weakly bound to the C‐terminal domain. To shrink the binding domain, we generated five additional fragments: GFP‐tagged N1 (1–50aa), GFP‐tagged N2 (51–103aa), GFP‐tagged N3 (104–173aa), GFP‐tagged N4 (1–103aa) and GFP‐tagged N5 (51–173aa). Co‐IP assays demonstrated that both GFP‐tagged N3 and GFP‐tagged N5 interacted with Flag‐tagged PIM2 (Fig. 2C). To determine which domain(s) of PIM2 bound to TTP, we fragmented PIM2 to create a GFP‐tagged N‐terminal domain, a GFP‐tagged kinase domain and a GFP‐tagged C‐terminal domain (Fig. 2D). Overexpressed HA‐tagged TTP interacted with the kinase domain of PIM2 (Fig. 2E). As shown in Fig. S1A, Flag‐tagged PIM2 (33‐286aa) also interacted with GFP‐tagged N3 (104‐173aa). Moreover, we also demonstrated that PIM2 kinase activity was dispensable for their interaction (Fig. S1B). Taken together, these results indicate that the tandem zinc finger domain of TTP and the kinase domain of PIM2 are critical for the interaction between the two proteins.

### 3.3 PIM2 negatively regulates TTP protein levels through the ubiquitin proteasome pathway

**Figure 3:**
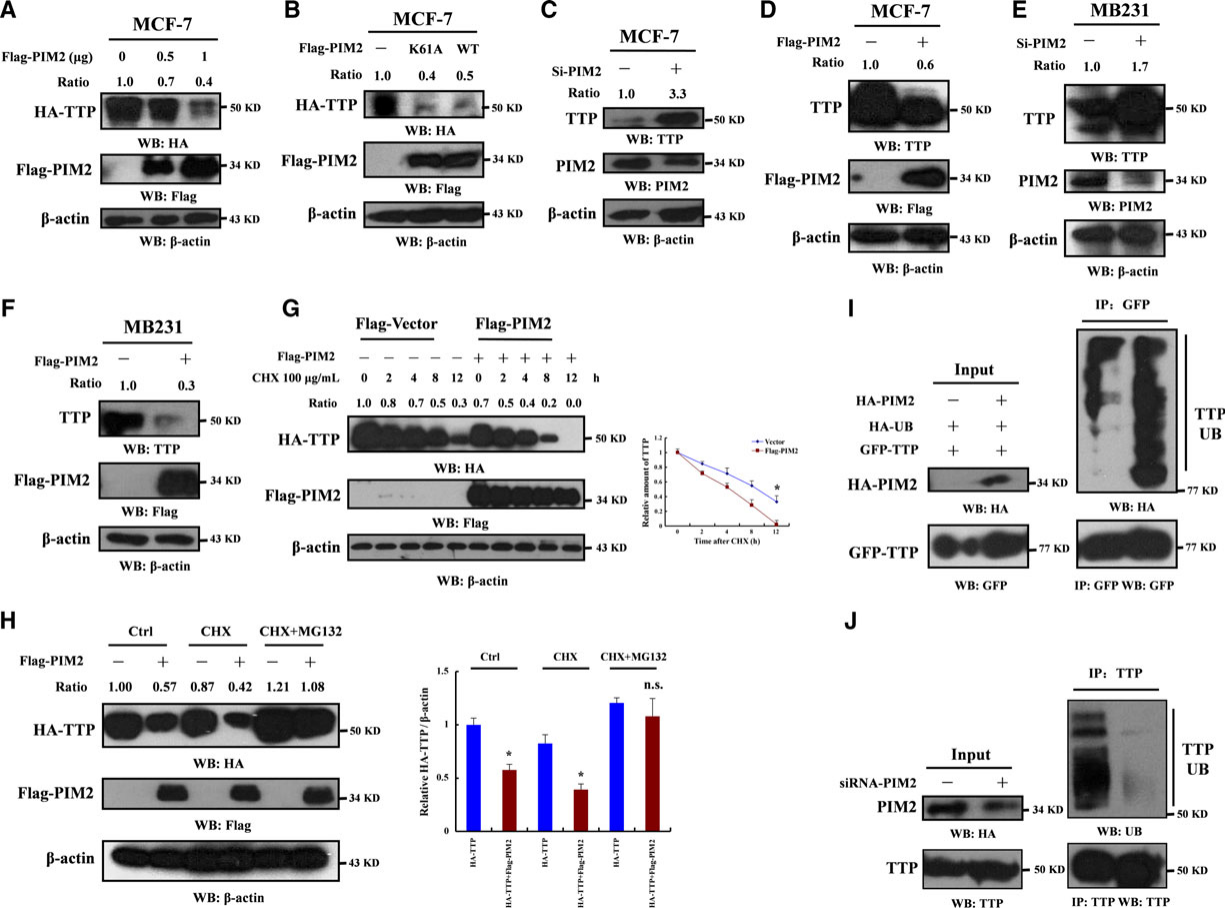
PIM2 negatively regulates TTP protein levels through the ubiquitin proteasome pathway. (A) MCF‐7 cells co‐transfected with HA‐tagged TTP and Flag‐tagged PIM2 (0, 0.5 or 1 μg) (empty vector as control). After 3 days of incubation, cell lysates were prepared and analyzed by western blotting using the indicated antibodies. The intensities of immunoblotting analyses were measured using imagej software (HA‐TTP normalized to β‐actin). (B) MCF‐7 cells co‐transfected with HA‐tagged TTP and Flag‐tagged PIM2 (kinase dead or wild type) (empty vector as control). Three days after transfection, the protein levels were analyzed by western blotting using the indicated antibody. The intensities of immunoblotting analyses were measured using imagej software (HA‐TTP normalized to β‐actin). (C) Effects of PIM2 knockdown by specific siRNA on endogenous TTP protein levels in MCF‐7 cells. Nonspecific (NS) siRNA was used as control. Three days after transfection, the protein levels were analyzed by Western blotting using the indicated antibody. The intensities of immunoblotting analyses were measured using imagej software (TTP normalized to β‐actin). (D) Effects of overexpression PIM2 on endogenous TTP protein levels in MCF‐7 cells. Empty vector was used as the control. Three days after transfection; the protein levels were analyzed by Western blotting using the indicated antibody. The intensities of immunoblotting analyses were measured using imagej software (TTP normalized to β‐actin). (E) Effects of PIM2 knockdown by specific siRNA on endogenous TTP protein levels in MDA‐MB231 cells. Nonspecific (NS) siRNA was used as the control. Three days after transfection, the protein levels were analyzed by western blotting using the indicated antibody. The intensities of immunoblotting analyses were measured using imagej software (TTP normalized to β‐actin). (F) Effects of overexpression PIM2 on endogenous TTP protein levels in MDA‐MB231 cells. Empty vector was used as the control. Three days after transfection, the protein levels were analyzed by western blotting using the indicated antibody. The intensities of immunoblotting analyses were measured using imagej software (TTP normalized to β‐actin). (G) MCF‐7 cells were co‐transfected with HA‐tagged TTP and Flag‐PIM2. Empty vector was used as control. After 2 days of incubation, cells were treated with 100 μg·mL^−1^ cycloheximide (CHX) for the indicated amount of time. The protein levels in cell lysates were analyzed by western blotting using the indicated antibody. The intensities of immunoblotting analyses were measured using imagej software (HA‐TTP normalized to β‐actin). Data are mean ± SD of three independent experiments, *P < 0.05. (H) MCF‐7 cells were co‐transfected with HA‐tagged TTP and Flag‐PIM2. Empty vector was used as control. Two days after transfection, cells were treated with CHX or MG132 or both for another 8 h of incubation. The proteins were detected by western blot using the indicated antibody. The intensities of immunoblotting analyses were measured using imagej software (HA‐TTP normalized to β‐actin). Data are mean ± SD of three independent experiments, *P < 0.05. (I) MCF‐7 cells were co‐transfected with HA‐tagged ubiquitin and GFP‐tagged TTP in the presence or absence of HA‐tagged PIM2. After 2 days of transfection, cells were treated with MG132 for another 8 h of incubation. The cell lysates were then immunoprecipitated with anti‐GFP antibody followed by western blotting using the indicated antibody. (J) MCF‐7 cells were transfected with PIM2‐specific siRNA in MCF‐7 cells. After 2 days of transfection, cells were treated with MG132 for another 8 h of incubation. The cell lysates were then immunoprecipitated with anti‐TTP antibody followed by western blotting using the indicated antibody.

PIM2 is a typical oncogene in many cancers, but TTP is reported as a tumor suppressor (Goddio *et al*., 2012; Swords *et al*., 2011). We therefore hypothesized that PIM2 might regulate TTP protein levels. We found that overexpressed Flag‐tagged PIM2 could dramatically decrease TTP protein levels in a dose‐dependent way (Fig. 3A). To examine whether PIM2 affects TTP protein levels independently of its kinase activity, we overexpressed kinase‐dead Flag‐tagged PIM2 (K61A) in MCF‐7 cells which had been proved kinase dead before (Yu *et al*., 2013). PIM2 (WT) decreased TTP protein levels compared with control, but the effect of PIM2 (K61A) on TTP was not different from PIM2 (WT), indicating that kinase activity was not required (Fig. 3B). To determine whether PIM2 directly phosphorylates TTP *in vitro*, we performed *in vitro* kinase assays. Our data demonstrated that PIM2 did not phosphorylate TTP *in vitro* (Fig. S2A,B), whereas it phosphorylated the known PIM2 kinase substrate PKM2 (Yu *et al*., 2013), under the same conditions (Fig. S2C). Moreover, knockdown of PIM2 by small‐interfering RNA increased exogenous TTP protein levels in MCF‐7 and MDA‐MB231 cells (Fig. 3C,E). Conversely, overexpression of Flag‐tagged PIM2 decreased exogenous TTP protein levels in MCF‐7 and MDA‐MB231 cells (Fig. 3D,F). We treated cells with cycloheximide (CHX) to test the half‐life of TTP. The overexpression of Flag‐tagged PIM2 markedly reduced the half‐life of TTP compared with overexpression of a control vector (Fig. 3G). Previous studies reported that TTP was degraded by the ubiquitin proteasome pathway (Ngoc *et al*., 2014). To determine whether the proteasome pathway regulates TTP stability, we transfected HA‐tagged TTP and Flag‐tagged PIM2 into MCF‐7 cells. Forty‐eight hours after transfection, we treated the cells with CHX or CHX + the specific proteasome inhibitor MG132 for 8 h, and tested the TTP protein levels by western blotting. MG132 inhibited the PIM2‐mediated degradation of TTP protein when protein synthesis was blocked by CHX (Fig. 3H). We performed an *in vivo* ubiquitination assay to test whether PIM2 affected TTP ubiquitination. Overexpression or knockdown of PIM2 could affect the TTP ubiquitination level in MCF‐7 cells (Fig. 3I,J). Those findings suggest that PIM2 negatively regulates TTP protein levels through the proteasome pathway.

### 3.4 PIM2 and TTP protein levels are negatively correlated in human breast cancer

**Figure 4:**
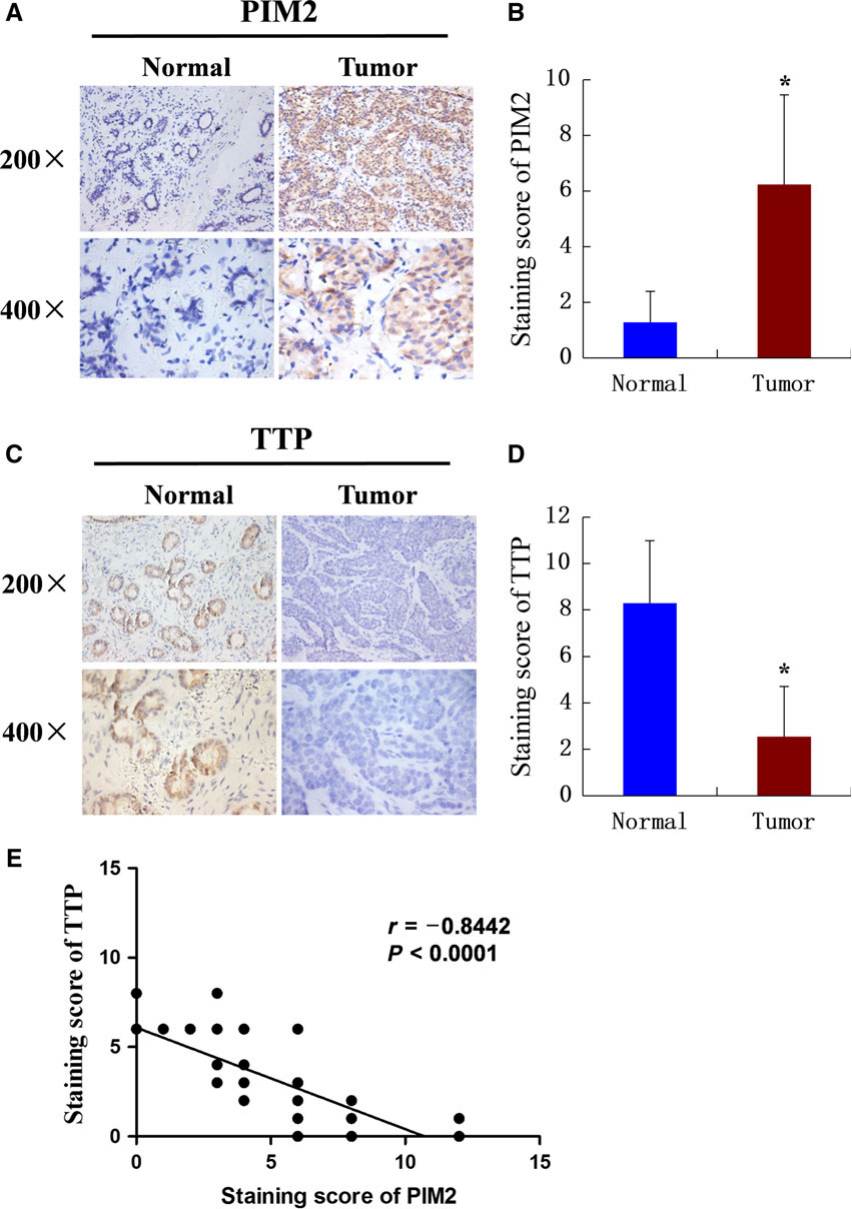
PIM2 and TTP protein levels are negatively correlated in human breast cancer. (A, C) Histopathologic sections of breast tissues were stained with anti‐PIM2 (A) or TTP (C) antibodies. Representative images of indicated breast tissues are shown (200×, top; 400×, bottom). (B, D) Semiquantitative analyses of immunohistochemistry data of human breast tissues for PIM2 (B) or TTP (D). The experiments were tested with paired *t*‐test, *P < 0.05. (E) Pearson correlative analysis of semiquantitative staining scores for PIM2 and TTP. The standard curve was drawn by linear regression of the correlation scores.

To determine the expressions of PIM2 and TTP in breast cancer tissues, we performed immunohistochemistry (IHC) assays in 84 cases of breast cancer tissues, and 10 cases of normal breast tissues as control. Immunohistochemical staining revealed that PIM2 was strongly expressed in breast cancer tissues (Fig. 4A,B); TTP, however, was expressed more in normal breast tissues (Fig. 4C,D). Consistently, we found that PIM2 protein levels were positively correlated with tumor size and stage of breast tumors, whereas TTP protein levels were negatively correlated (Table 1). We also determined the correlation between PIM2 and TTP expression in breast cancer (Fig. 4E). These data show that the protein levels of PIM2 and TTP are negatively correlated in human breast cancer.

**Table 1:** Analysis of correlation between PIM2 and TTP protein levels and clinicopathological parameters of breast cancer patients

Variable	PIM2 expression			*P‐*value	TTP expression			*P*‐value
	*n*	Low	High		*n*	Low	High	
Age								
≤ 50 years	29	10	19	0.292	29	23	6	0.941
> 50 years	55	13	42		55	44	11	
Tumor size								
≤ 2 cm	36	19	17	< 0.0001	36	23	13	0.002
> 2 cm	48	4	44		48	44	4	
TNM stage								
I–II	49	19	30	0.006	49	35	14	0.025
III–IV	35	4	31		35	32	3	
ER status								
+	43	12	31	0.912	43	37	6	0.144
‐	41	11	30		41	30	11	
PR status								
+	50	15	35	0.516	50	42	8	0.244
‐	34	8	26		34	25	9	
HER2								
+	38	13	25	0.205	38	29	9	0.478
‐	46	10	36		46	38	8	

### 3.5 PIM2 overexpression leads to decrease mRNA levels of TTP‐associated transcripts

**Figure 5:**
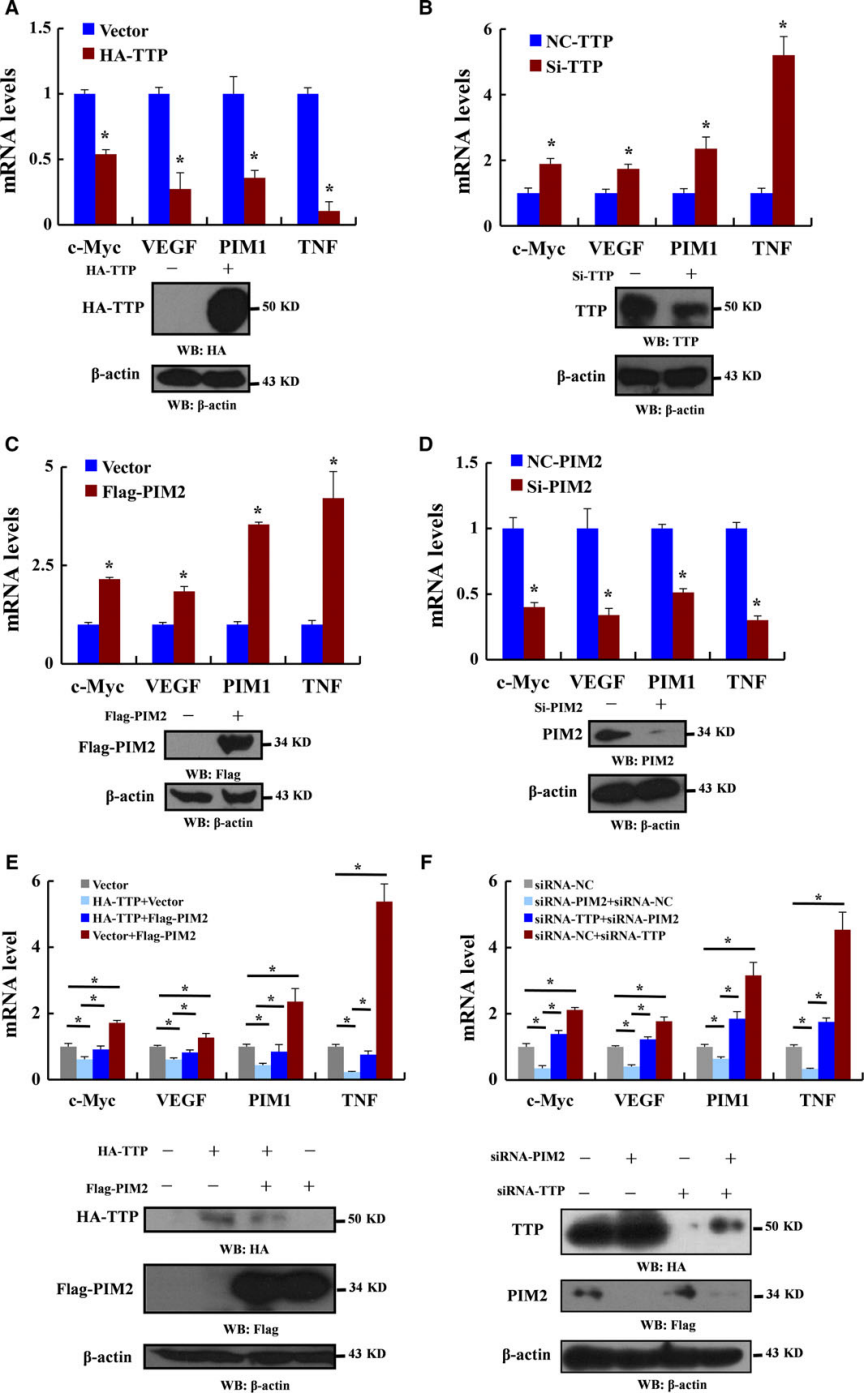
Overexpression of PIM2 leads to a decrease in mRNA levels of TTP‐associated transcripts. (A) MCF‐7 cells were transfected with HA‐tagged TTP and empty vector as control. Three days after transfection, the protein levels were detected by western blotting using the indicated antibodies, and qRT‐PCR was performed to analyze TTP‐targeted mRNA levels. (B) MCF‐7 cells were transfected with TTP‐specific siRNA and nonspecific (NS) siRNA as control. Three days after transfection, the protein levels were detected by western blotting using the indicated antibodies, and qRT‐PCR was performed to analyze TTP‐targeted mRNA levels. (C) MCF‐7 cells were transfected with Flag‐tagged PIM2 and empty vector as control. Three days after transfection, the protein levels were detected by western blotting using the indicated antibodies, and qRT‐PCR was performed to analyze TTP‐targeted mRNA levels. (D) MCF7 cells were transfected with PIM2‐specific siRNA and nonspecific (NS) siRNA as control. Three days after transfection, the protein levels were detected by western blotting using the indicated antibodies, and qRT‐PCR was performed to analyze TTP‐targeted mRNA levels. (E) MCF‐7 cells were transfected with HA‐tagged TTP or Flag‐tagged PIM2 or both. The protein levels were detected by western blotting using the indicated antibodies. Three days after transfection, qRT‐PCR was performed to analyze TTP‐targeted mRNA levels. (F) MCF‐7 cells were transfected with TTP or PIM2‐specific siRNA or both. The protein levels were detected by western blotting using the indicated antibodies. Three days after transfection, qRT‐PCR was performed to analyze TTP‐targeted mRNA levels. All data are the mean ± SD of three independent experiments, *P < 0.05.

Our earlier study suggested that PIM2 could accelerate TTP degradation, so we tested whether that association impaired TTP functions. To determine TTP‐mediated mRNA transcripts, we overexpressed HA‐tagged TTP or knocked‐down TTP in MCF‐7 cells. TTP overexpression reduced the mRNA levels of its target genes, including c‐MYC, VEGF, PIM1 and tumor necrosis factor (Fig. 5A), and knockdown of TTP increased mRNA levels of its target genes (Fig. 5B). There was no marked change in the level of TTP nontarget gene‐PIM2 mRNA (Kim *et al*., 2012; Selmi *et al*., 2012) (Fig. S3A). We studied whether PIM2 affected mRNA levels of TTP target genes. PIM2 significantly increased the mRNA levels of TTP target genes (Fig. 5C,D). Interestingly, PIM2 (K61A) kinase dead mutant could also increase expression of TTP target genes (Fig. S3B), which demonstrated that PIM2‐mediated TTP target genes expression was independent of its kinase activity. Furthermore, PIM2 was able to abolish TTP‐mediated mRNA levels (Fig. 5E,F). Thus, our data reveal that PIM2 abrogates TTP‐associated transcripts.

### 3.6 PIM2 regulates TTP‐mediated proliferation and migration in breast cancer cells

**Figure 6:**
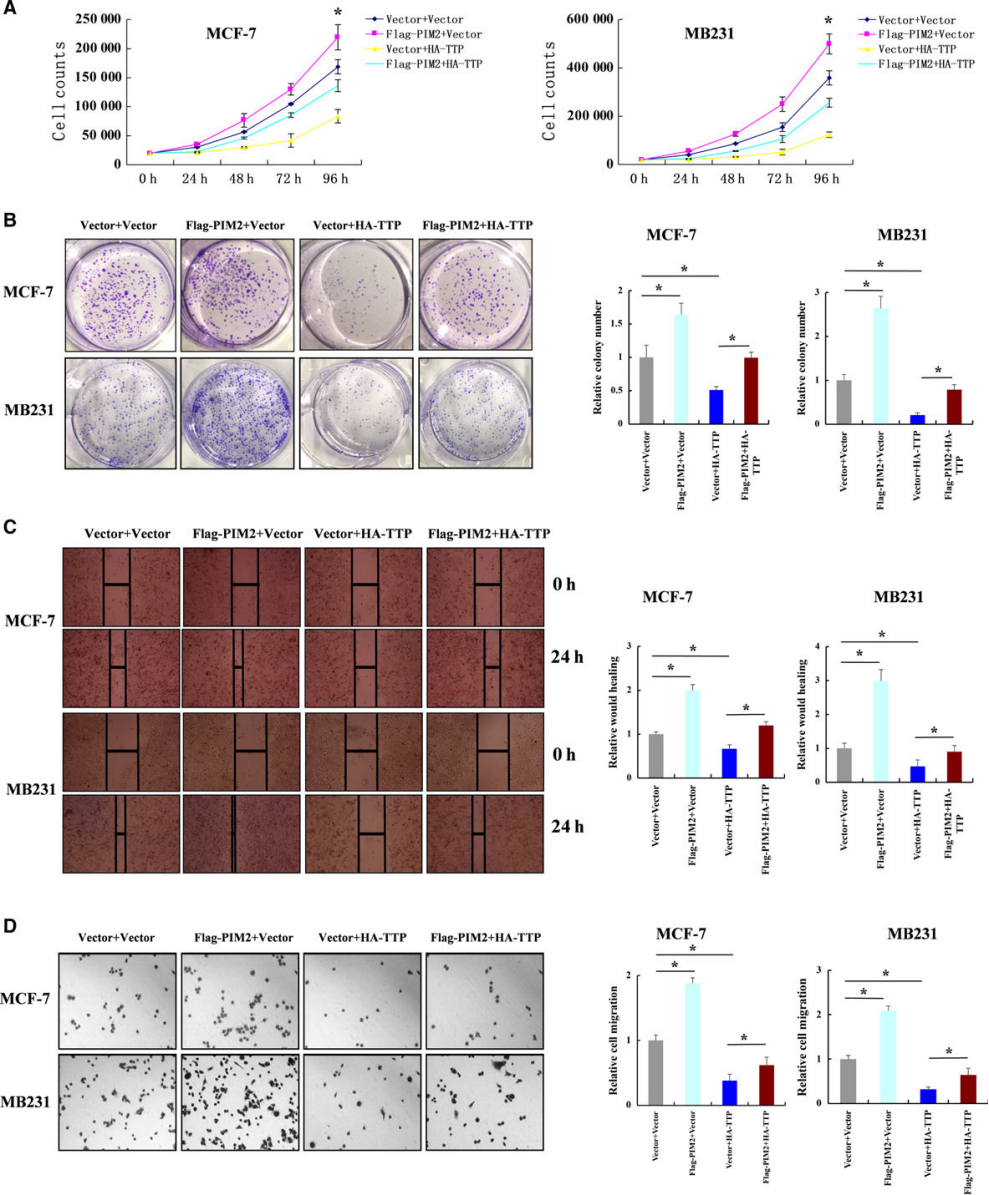
PIM2 regulates TTP‐mediated cell proliferation and migration in breast cancer cells. (A) MCF‐7 or MDA‐MB231 cells were transfected with Flag‐tagged PIM2, HA‐tagged TTP or both HA‐tagged TTP and Flag‐tagged PIM2 (empty vector as control). One day after transfection, cells were re‐plated and analyzed for cell growth by counting cell numbers at the indicated time points. (B) MCF‐7 or MDA‐MB231 cells were transfected with Flag‐tagged PIM2, HA‐tagged TTP or both (empty vector as control). One day after transfection, cells were re‐plated with agarose gel to perform colony formation assays. (C) MCF‐7 or MDA‐MB231 cells were transfected with either Flag‐tagged PIM2 or HA‐tagged TTP, or both (empty vector as control). One day after transfection, cells were re‐plated to perform scratch test assays. Cell migration was determined after 1 day. Representative images (100×). (D) MCF‐7 or MDA‐MB231 cells were transfected with either Flag‐tagged PIM2 or HA‐tagged TTP, or both (empty vector as control). One day after transfection, cells were re‐plated to perform transwell assays. Cell numbers were counted for the analysis of cell migration after 12 h. Representative images (100×). All data are mean ± SD of three independent experiments, *P < 0.05.

To determine whether PIM2 regulates the effect of TTP on cell proliferation, we overexpressed HA‐tagged TTP and Flag‐tagged PIM2 in breast cancer cells. PIM2 overexpression significantly decreased TTP‐mediated inhibition of cell proliferation (Fig. 6A,B). Moreover, cell migration was critical for breast cancer development. Cell‐scratch tests and transwell migration assays indicated that PIM2 decreased TTP‐mediated inhibition of cell migration (Fig. 6C,D). These results show that PIM2 is important in the regulation of TTP‐mediated proliferation and migration in breast cancer cells.

### 3.7 PIM2‐mediated TTP function promotes breast tumor growth *in vivo*

**Figure 7:**
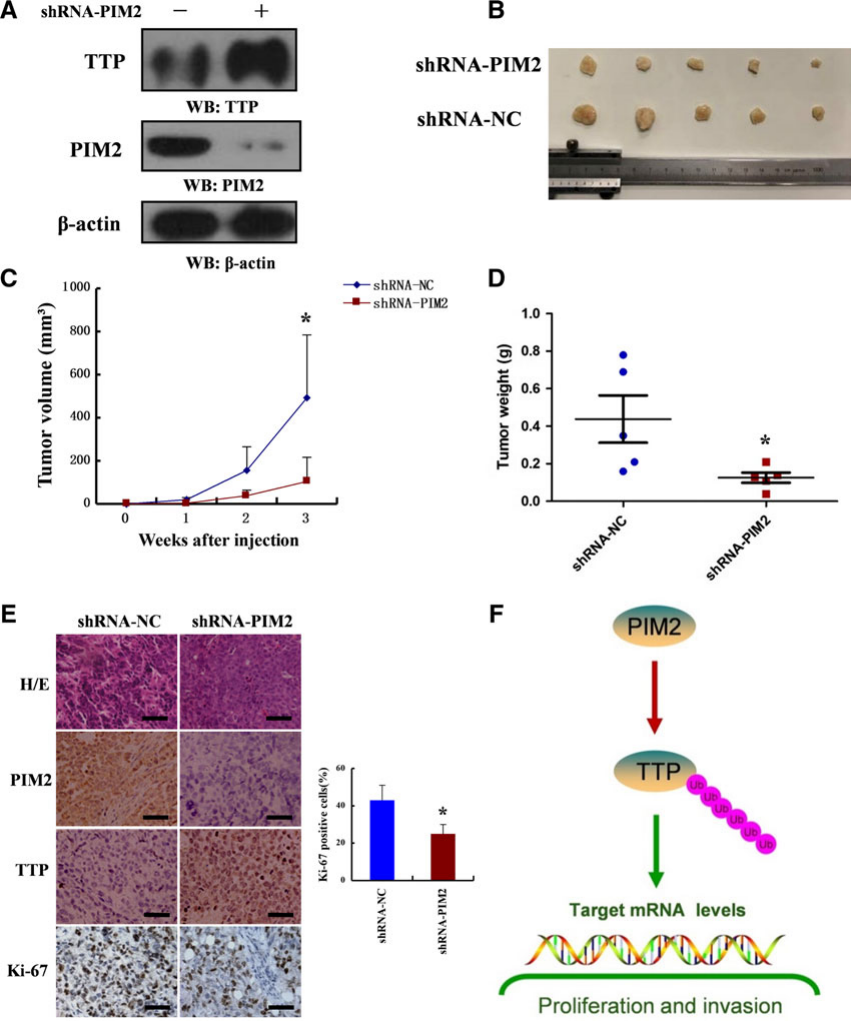
PIM2‐mediated TTP function promotes breast tumor growth *in vivo*. (A) shRNA‐PIM2 was stably expressed in MCF‐7 cells followed by western blotting using indicated antibodies. (B) Photographs of tumors excised 3 weeks after inoculation of stably transfected cells into nude mice. (C) Tumor volumes were measured during the tumor growth for 3 weeks. Tumor volumes were calculated according to the following formula: Tumor volume = (length × width^2^)/2. (D) After 3 weeks, the nude mice were killed and tumor weights were measured. (E) Nude mice tumor tissues were paraffin‐embedded and tumor slides were stained with hematoxylin & eosin (H&E), antibodies of PIM2, TTP and Ki‐67 (400×). (F) Schematic diagram of the proposed PIM2 regulation of TTP degradation in breast cancer. All data are the mean ± SD of five independent experiments, *P < 0.05.

To determine whether the PIM2‐mediated TTP regulates breast tumor growth *in vivo*, we performed xenograft studies. Stable transfection with shRNA‐PIM2 increased TTP protein levels in MCF‐7 cells (Fig. 7A). Tumors generated by subcutaneous implantation in nude mice were monitored every week after injection. After 3 weeks, tumors were removed from the mice (Fig. 7B). The tumors in the shRNA‐PIM2 cells grew significantly slower than those in the shRNA‐NC cells (Fig. 7C). The weight of tumors in the shRNA‐PIM2 cells was substantially lighter as compared with the shRNA‐NC cells (Fig. 7D). Stable knockdown of PIM2 strongly increased immunostaining of TTP in grafted cells (Fig. 7E). Using Ki‐67 staining as a measure of cell proliferation we further confirmed that shRNA‐PIM2 cells were less proliferative than shRNA‐NC cells *in vivo* (Fig. 7E). These results suggest that PIM2‐mediated TTP function confers a tumor cell growth advantage *in vivo*.

## 4 Discussion

PIM2 is an oncogene that regulates many signal pathways in cancer. PIM2 phosphorylates many substrates to change their functions, thereby playing an important role in tumorigenesis (Jinesh *et al*., 2016). PIM2 is upregulated in multiple cancer types and promotes cancer‐cell survival by unknown mechanisms. We used multiple biochemical approaches to demonstrate that PIM2 directly bound to TTP *in vivo* and *in vitro*. In addition, PIM2 regulated TTP‐reduced cell proliferation and migration, implying that the PIM2–TTP complex plays a significant role in the progression of human breast tumors. Although PIM2 is a serine/threonine kinase, it regulates TTP functions independently of its kinase activity, consistent with our previous study (Yu *et al*., 2014b). Moreover, our data demonstrated that kinase dead mutant PIM2 (K61A) could still promote cell migration compared with the empty vector (Fig. S4). Thus PIM2 might affect the functions of other proteins not only because of its kinase activity but also by physically binding. Therefore, as a target for cancer treatment, PIM2 knockdown might be more efficient than the drug‐induced inhibition of PIM2 kinase activity.

The proto‐oncogene PIM family has three members: PIM1, PIM2 and PIM3. They constitute a distinct class of protein kinases with a specificity towards phosphorylation on serine/threonine residues (Saris *et al*., 1991). In recent reports, PIM1 kinase played an important role in regulating triple‐negative breast cancer (Braso‐Maristany *et al*., 2016; Horiuchi *et al*., 2016) and PIM2‐specific siRNA treatment was as effective as PIM1‐specific siRNA treatment in inhibiting breast cell proliferation, which was more effective in inducing cell death (Horiuchi *et al*., 2016). Moreover, PIM2 overexpression in mice could promote breast cancer tumorigenesis (Jimenez‐Garcia *et al*., 2017). But the mechanisms of PIM2 by which regulates breast cancer cell proliferation are still unclear, Our data showed that PIM2 was crucial in the regulation of TTP‐reduced proliferation and migration in breast cancer cells, consistent with previous studies (Horiuchi *et al*., 2016).

Previous studies reported that TTP regulated the mRNA decay of PIM1 and PIM3 but not that of PIM2 (Kim *et al*., 2012; Mahat *et al*., 2012; Selmi *et al*., 2012). Our study showed a negative feedback loop between PIM family proteins and TTP through the PIM2‐degradation of TTP. The subcellular localization of TTP affected the functions of TTP in cancer (Fairhurst *et al*., 2003). TTP proteins mostly distributed in the cytoplasm to decay mRNA, but nuclear TTP proteins had other important functions to regulate tumor progression. For example, nuclear TTP suppressed the transcriptional activity of nuclear factor kappa B (NF‐κB) and a lack of TTP led to an increase in nuclear p65 protein levels, which is essential for inflammation (Schichl *et al*., 2009). Furthermore, nuclear TTP modulated the transactivation activity of progesterone receptor (PR), glucocorticoid receptor (GR) and androgen receptor (AR) and acted as a co‐repressor of PR, GR and AR in breast cancer (Barrios‐Garcia *et al*., 2016). Interestingly, we found PIM2 bound to the nuclear localization‐signal domain of TTP, therefore how PIM2 regulated TTP localization might need to be investigated further.

## 5 Conclusions

In conclusion, we identified PIM2 as a new regulator of TTP. Our results showed that PIM2 and TTP interacted with each other. Functionally, PIM2 regulated several aspects of TTP functions in the reprogramming of cancer cells through protein degradation and protein binding. Our results demonstrated that PIM2‐mediated TTP degradation was crucially involved in the regulation of proliferation and migration in breast cancer cells (Fig. 7F). Our results suggest that the PIM2 is a potential therapy target for the treatment of breast cancer.

## Author contributions

ZY and CR designed research. TY, ZY, CR, QP and LW performed research. XH, YD, SL, ZL and YS contributed new reagents/analytic tools. ZY and CR analyzed data. ZY wrote and revised the paper.

## Supporting information


**Fig. S1.** PIM2 kinase activity is dispensable for PIM2‐TTP complex. (A) MCF‐7 cells were co‐transfected with GFP‐tagged TTP (104‐173) and Flag‐tagged PIM2 (33‐286). After 24 h in culture, cell lysates were prepared in IP buffer and Flag‐tagged proteins were immunoprecipitated with anti‐Flag antibody (IgG as control). The proteins were examined by western blotting using the indicated antibodies. (B) MCF‐7 cells were co‐transfected with HA‐tagged TTP and Flag‐tagged PIM2 (kinase dead). After 24‐h culture, cell lysates were prepared in IP buffer and HA‐tagged proteins were immunoprecipitated with anti‐HA antibody (IgG as control). The proteins were examined by western blotting using the indicated antibodies.

**Fig. S2.** TTP is not phosphorylated by PIM2 *in vitro*. (A) I*n vitro* kinase assay was used to determine the effects of recombinant PIM2 on serine phosphorylation of GST‐tagged TTP. (B) *In vitro* kinase assay was used to determine the effects of recombinant PIM2 on threonine phosphorylation of GST‐tagged TTP. (C) In vitro kinase assay was used to determine the effects of recombinant PIM2 on threonine phosphorylation of GST‐tagged PKM2.

**Fig. S3.** PIM2 (K61A) kinase dead mutant also increases TTP‐mediated mRNA levels. (A) MCF‐7 cells were transfected HA‐tagged TTP or TTP‐specific siRNA. Three days after transfection, qRT‐PCR was performed to analyze PIM2 mRNA levels. (B) MCF‐7 cells were transfected with Flag‐tagged PIM2 (K61A or WT) and empty vector as control. Three days after transfection, the protein levels were detected by western blotting using the indicated antibodies, and qRT‐PCR was performed to analyze TTP‐targeted mRNA levels. All data are the mean ± SD of three independent experiments, *P < 0.05.

**Fig. S4.** PIM2 (K61A) kinase dead mutant still promotes cell migration in breast cancer cells. MCF‐7 or MDA‐MB231 cells were transfected with Flag‐tagged PIM2 (K61A or WT) and empty vector as control. One day after transfection, cells were re‐plated to perform transwell assays. Cell numbers were counted for the analysis of cell migration after 12 h. Representative images (100+). Data are the mean ± SD of three independent experiments, *P < 0.05.

**Table S1.** Interaction proteins with TTP.

**Table S2.** Materials and primers.
