# Supplementary figures and images for: PIM2 interacts with tristetraprolin and promotes breast cancer tumorigenesis
Source: Mol Oncol. 2018 Apr 14;12(5):690–704. doi: 10.1002/1878-0261.12192 (PMC5928357; doi:10.1002/1878-0261.12192)

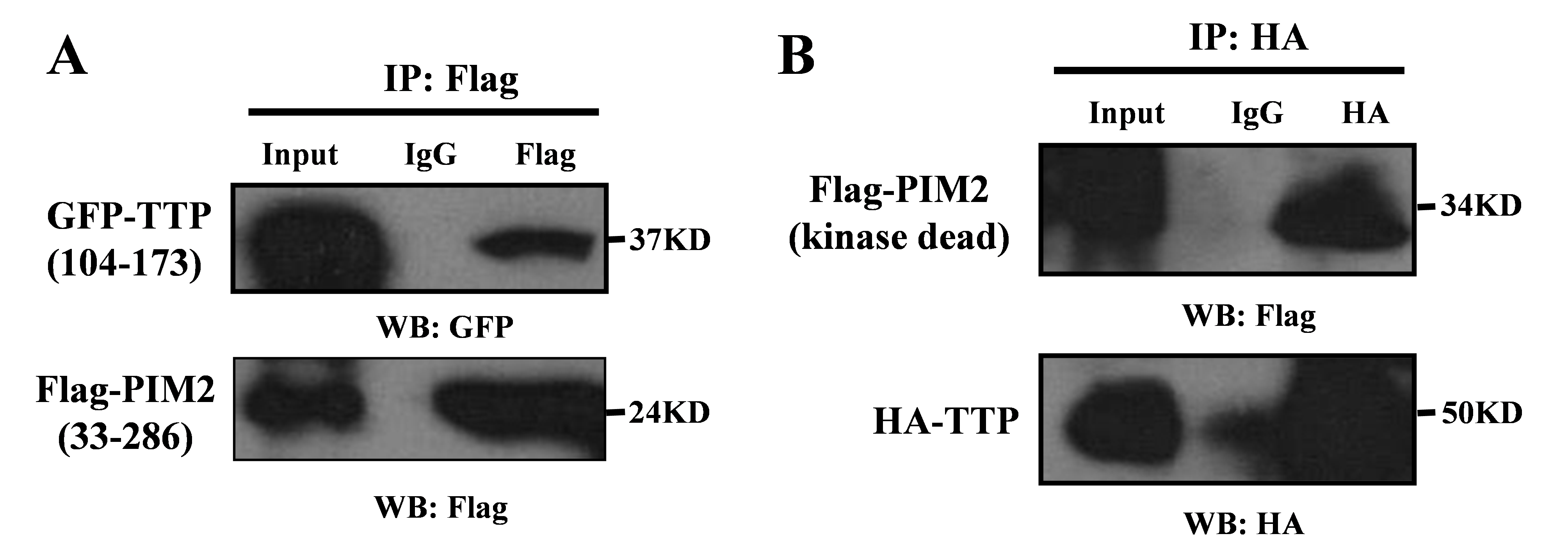

Supplement: Supplementary file 1 — Fig. S1. PIM2 kinase activity is dispensable for PIM2‐TTP complex. (A) MCF‐7 cells were co‐transfected with GFP‐tagged TTP (104‐173) and Flag‐tagged PIM2 (33‐286). After 24 h in culture, cell lysates were prepared in IP buffer and Flag‐tagged proteins were immunoprecipitated with anti‐Flag antibody (IgG as control). The proteins were examined by western blotting using the indicated antibodies. (B) MCF‐7 cells were co‐transfected with HA‐tagged TTP and Flag‐tagged PIM2 (kinase dead). After 24‐h culture, cell lysates were prepared in IP buffer and HA‐tagged proteins were immunoprecipitated with anti‐HA antibody (IgG as control). The proteins were examined by western blotting using the indicated antibodies. [file MOL2-12-690-s001.tif]

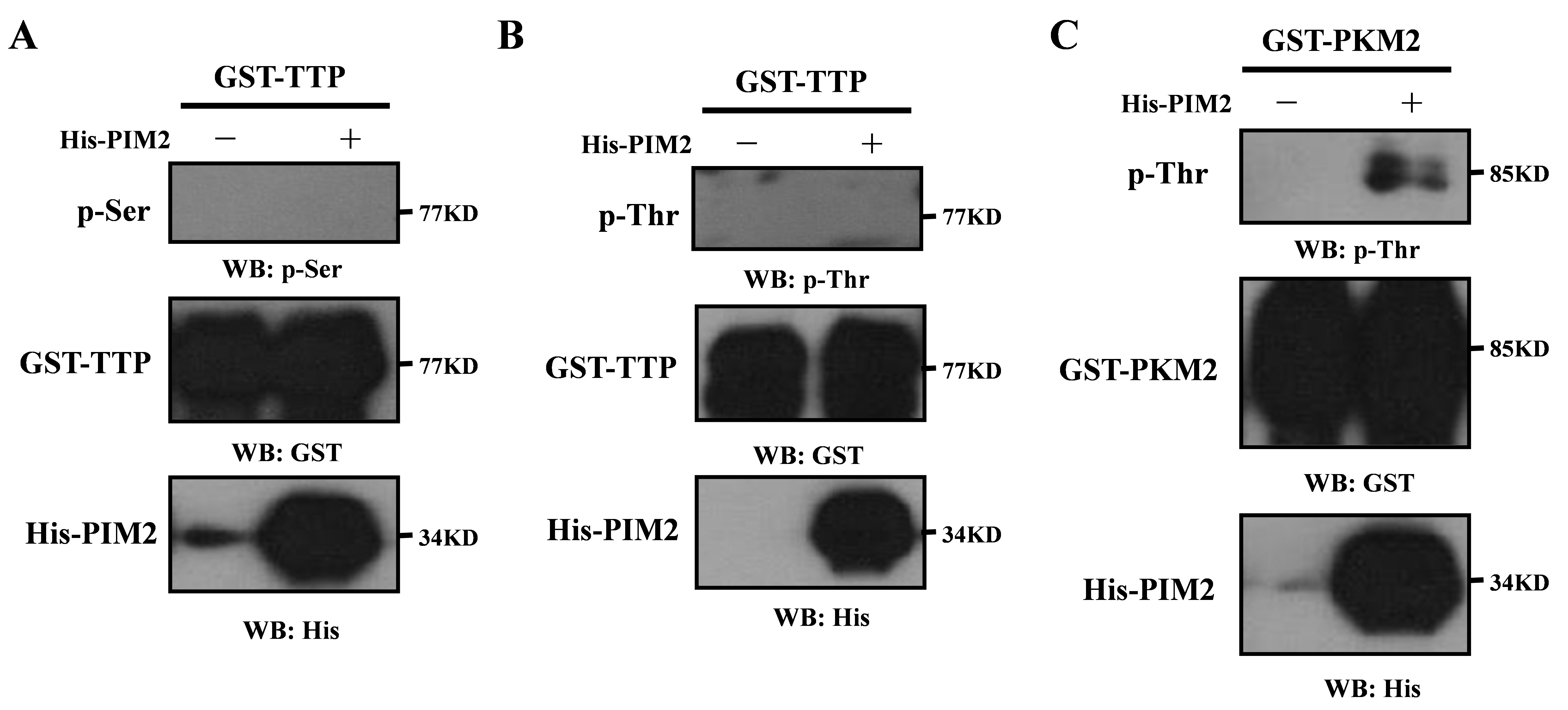

Supplement: Supplementary file 2 — Fig. S2. TTP is not phosphorylated by PIM2 in vitro. (A) In vitro kinase assay was used to determine the effects of recombinant PIM2 on serine phosphorylation of GST‐tagged TTP. (B) In vitro kinase assay was used to determine the effects of recombinant PIM2 on threonine phosphorylation of GST‐tagged TTP. (C) In vitro kinase assay was used to determine the effects of recombinant PIM2 on threonine phosphorylation of GST‐tagged PKM2. [file MOL2-12-690-s002.tif]

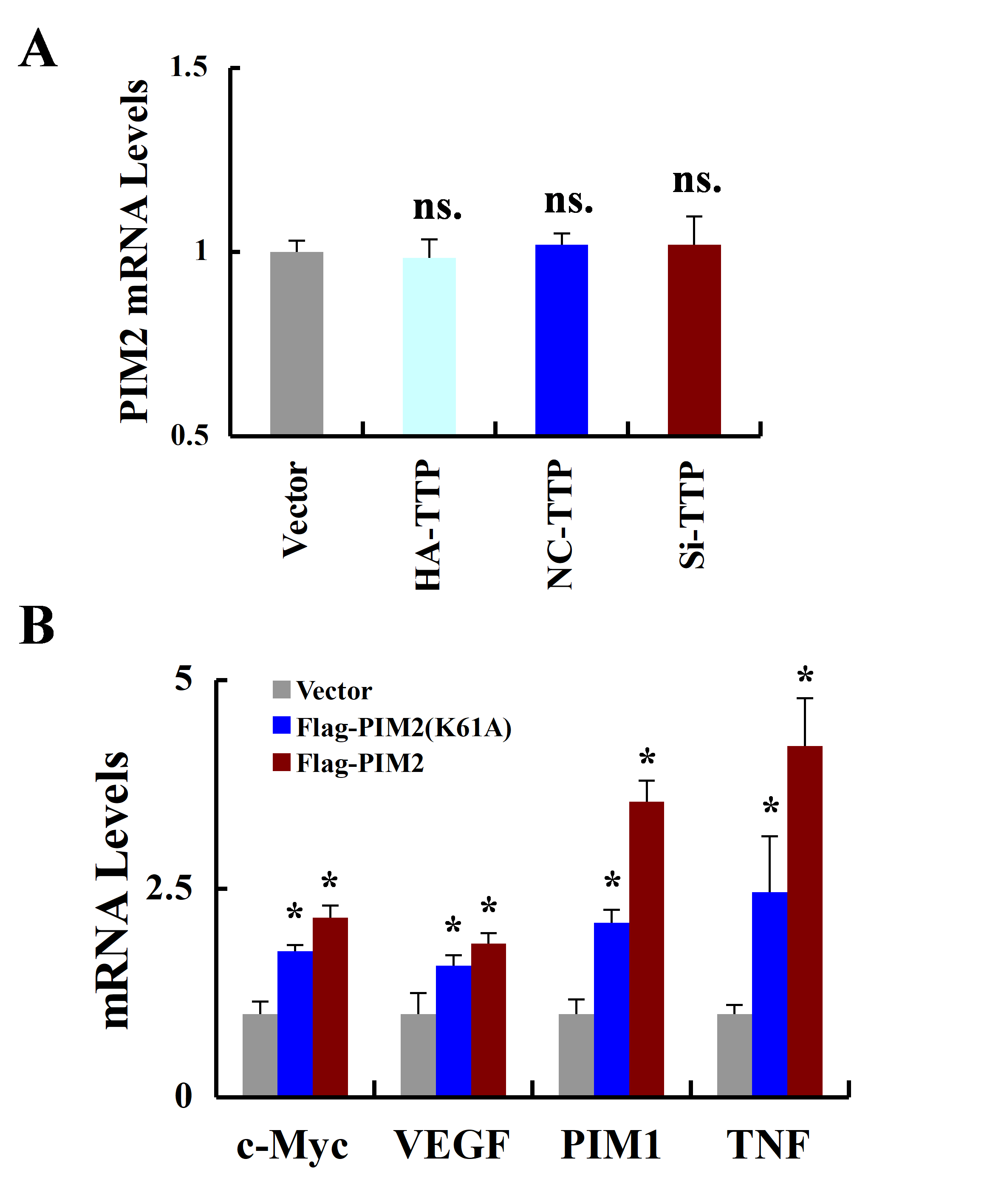

Supplement: Supplementary file 3 — Fig. S3. PIM2 (K61A) kinase dead mutant also increases TTP‐mediated mRNA levels. (A) MCF‐7 cells were transfected HA‐tagged TTP or TTP‐specific siRNA. Three days after transfection, qRT‐PCR was performed to analyze PIM2 mRNA levels. (B) MCF‐7 cells were transfected with Flag‐tagged PIM2 (K61A or WT) and empty vector as control. Three days after transfection, the protein levels were detected by western blotting using the indicated antibodies, and qRT‐PCR was performed to analyze TTP‐targeted mRNA levels. All data are the mean ± SD of three independent experiments, *P < 0.05. [file MOL2-12-690-s003.tif]

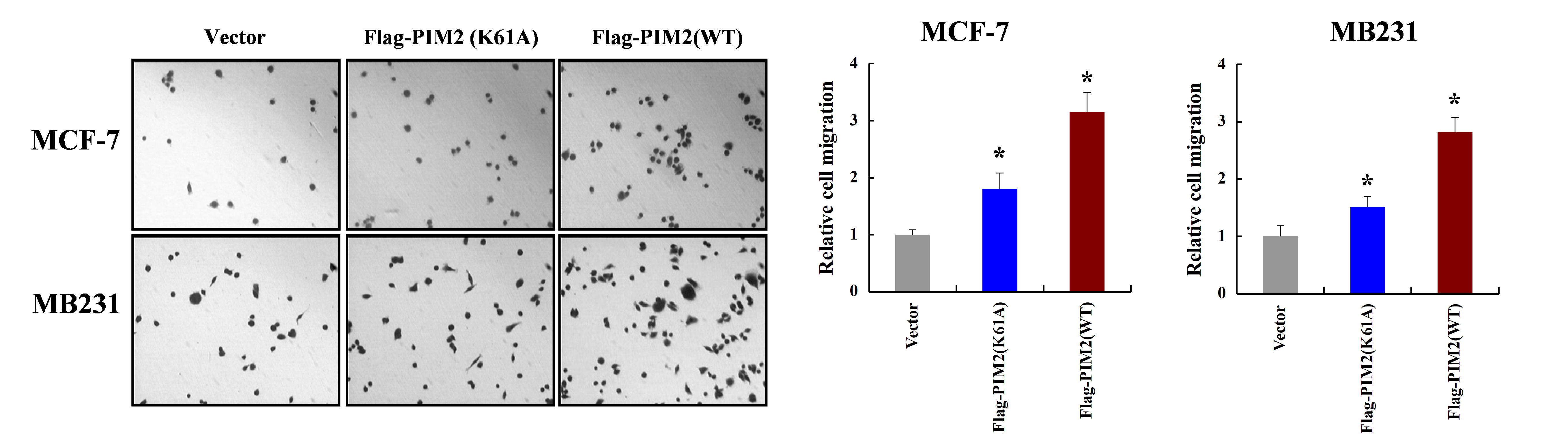

Supplement: Supplementary file 4 — Fig. S4. PIM2 (K61A) kinase dead mutant still promotes cell migration in breast cancer cells. MCF‐7 or MDA‐MB231 cells were transfected with Flag‐tagged PIM2 (K61A or WT) and empty vector as control. One day after transfection, cells were re‐plated to perform transwell assays. Cell numbers were counted for the analysis of cell migration after 12 h. Representative images (100+). Data are the mean ± SD of three independent experiments, *P < 0.05. [file MOL2-12-690-s004.tif]
