# Supplementary material for: PIM2 interacts with tristetraprolin and promotes breast cancer tumorigenesis
Source: Mol Oncol. 2018 Apr 14;12(5):690–704. doi: 10.1002/1878-0261.12192 (PMC5928357; doi:10.1002/1878-0261.12192)
Supplement: Supplementary file 6 — Table S2. Materials and primers. [file MOL2-12-690-s006.doc]

**Table S1. Materials used in this research**

| **siRNAs used in this research** | |
| --- | --- |
| NC-siRNA | 5' UUCUCCGAACGGUCACGU 3’ |
| PIM2-siRNA | 5' CUCGAAGUCGCACUGCUAU 3’ |
| TTP-siRNA | 5' CGCUGCCACUUCAUCCACAAC 3’ |
| **Primer sequences used in qRT-PCR assays** | |
| MYC | Fw: 5' GAAGGGCAGGGCTTCTCAGAGGCTT 3’  Rev: 5' TATTCGCTCCGGATCTCCCTTCCC 3’ |
| VEGF | Fw: 5' CTTGCCTTGCTGCTCTAC 3’  Rev: 5' TGGCTTGAAGATGTACTCG 3’ |
| PIM1 | Fw: 5’ CAGCAGCAGCAGCAGCAACCACTA 3’  Rev: 5’ TTGGTGGCGTGCAGGTCGTT 3’ |
| TNF | Fw: 5’ AGACGCTCCCTCAGCAAGGA 3’  Rev: 5’ TCCCGGATCATGCTTTCAGT 3’ |
| PIM2 | Fw: 5’-ATGTTGACCAAGCCTCTACA-3’  Rev: 5’-TGACTGAGTCTGACAAGGGG-3’ |
| 18S | Fw: 5’ GTTGAACCCCATTCGTGATG 3’  Rev: 5’ GCCTCACTAAACCATCCAA 3’ |
| **Antibodies and materials used in this research** | |
| anti-PIM2 antibody | Genetex #GTX113928 |
| anti-TTP antibody | Genetex # GTX83404  Sigma #T5452 |
| anti-HA antibody | Sigma #H9658 |
| anti-GFP antibody | Sigma #G6539 |
| anti-Flag antibody | Sigma #F1804 |
| anti-β-actin antibody | Sigma #A5441 |
| anti-GST antibody | Sigma #G1160 |
| anti-His antibody | Sigma # SAB2702218-100UL |
| Normal IgG | Santa cruz biotechnology #sc-2025  Santa cruz biotechnology #sc-2027 |
| Anti-Phospho-threonine antibody | CST # 9386 |
| Anti-Phospho-serine antibody | Abcam #ab6639 |
| Goat anti-Mouse second antibody | Proteintech # SA00001-1 |
| Goat anti-Rabbit second antibody | Proteintech #SA00001-2 |
| Histostain-SP (Streptavidin-Peroxidase)kit | Bioss # SP-0023 |
| MG132 | Sigma # M7449-200UL |
| Cycloheximide | Genetex #GTX47936 |
| Protein A agarose | Pierce #2033 |
| Glutathione Sepharose 4B | GE healthcare life science # 17075601 |
| Ni Sepharose 6 Fast Flow | GE healthcare life science # 17-5318-01 |
| Enhanced chemoluminescent autoradiography | Thermo Fisher Scientific #34080 |
